# Being a nursing student during the coronavirus pandemic: a mixed methods study

**DOI:** 10.1186/s12912-023-01218-8

**Published:** 2023-03-03

**Authors:** Gudrun Rohde, Berit Johannessen, Markus Maaseide, Sylvi Flateland, Anne Valen Skisland, Ellen Benestad Moi, Kristin Haraldstad

**Affiliations:** 1 Department of Health and Nursing, Faculty of Health and Sport Sciences, University of Agder, Postbox 422, 4604 Kristiansand, Norway; 2 Department of Clinical Research, Sorlandet Hospital, Kristiansand, Norway

**Keywords:** Fear of COVID-19, Quality of life, General health, Nursing students, Mixed methods

## Abstract

**Background:**

The COVID-19 pandemic led to major changes in people’s lives via protective strategies aimed at limiting the transmission of COVID-19, including social distancing, lockdowns, cancelled or limited leisure activities and tutorials and supervision for students taking place digital. All of these changes may have influenced students’ health and quality of life.

**Aim:**

To describe and explore fear of COVID-19 and psychological distress, as well as general health and quality of life, among baccalaureate nursing students at 1 year into the COVID-19 pandemic.

**Method:**

We used a mixed method study design, including quantitative data from University of Agder, data that was a part of a national survey of baccalaureate nursing students nearly one year into the pandemic. All the nursing students at the university were invited to take part between 27 January and 28 February 2021. The quantitative survey included 396 (out of total 858) baccalaureate nursing students (response rate: 46%). The quantitative data were collected using well-validated measures of fear of COVID-19, psychological distress, general health and quality of life, and the data were analysed using the ANOVA-tests for continuous data and chi-square tests for categorical data. Qualitative data were gathered from focus group interviews from the same university two-three months later. Five focus group interviews were conducted with a total of 23 students (7 men, 16 women). The qualitative data were analysed using systematic text condensation.

**Results:**

The mean score (standard deviation [SD]) for fear of COVID-19 was 2.32 (0.71), for psychological distress was 1.53 (1.00), for general health was 3.51 (0.96) and for overall quality of life was 6.01 (2.06). In the qualitative data, we identified the overarching theme *effect of COVID-19 on students’ quality of life* and the three main themes; importance of personal relations, physical health challenges and mental health challenges.

**Conclusion:**

The COVID-19 pandemic influenced negatively nursing students’ quality of life and physical and mental health, and they often felt lonely. However, most of the participants also adapted strategies and resilience factors to cope with the situation. Via the pandemic situation, the students learned additional skills and mental mindsets that may be useful in their future professional lives.

**Supplementary Information:**

The online version contains supplementary material available at 10.1186/s12912-023-01218-8.

## Introduction

The United Nations third Sustainable Development Goal is to ensure healthy lives and to promote quality of life (QOL) for all people of all ages [1]. This goal has been incorporated into national guidelines and goals, healthcare practices and public awareness. QOL is increasingly used as an outcome measure in different research settings, including clinical practice and student population surveys. In this context, QOL is defined as individuals’ perception of their position in life in the context of the culture in which they live and in relation to their goals, expectations, standards and concerns [2]. When the COVID-19 pandemic hit in March 2020, it led to major changes in people’s lives through protective strategies aimed at limiting the transmission of COVID-19. Such strategies included social distancing, lockdowns, cancelled or limited leisure activities and digital rather than in-person tutorials for students [3–5], which may all have influenced people’s health and QOL.

The consequences of COVID-19 restrictions and lockdown especially influenced young people and students. Nursing students may have been particularly vulnerable during the COVID-19 pandemic because their practical and clinical training during their nursing programmes [5–8]. Previous studies on nursing students during the COVID-19 pandemic revealed that stress increased substantially during lockdown. Stress and anxiety could be triggered by students’ personal problems [8–10]. Studies also showed that during the pandemic nursing students experienced loneliness and mental health problems, sleeplessness, problems with concentration and learning efficiency and fear of infection, as well as anxiety regarding grades, passing exams and final graduation [10–13].

Statistics indicate that Norwegian society has handled the COVID-19 pandemic rather well. Most inhabitants have followed governmental rules, the transmission rate has been low and there have been relatively few hospital admissions and few deaths compared with the rates in other countries [14]. However, adolescents and young adults have seemed to suffer, and COVID-19 restrictions may have had negative effects on their QOL [15]. In a study of QOL among American nursing students, the results show that the students reported poorer QOL during the COVID-19 pandemic, specifically in the areas of psychological health and social relationships [16]. In a survey administered during the second wave of the pandemic (January – March 2021), Norwegian baccalaureate nursing students reported worse outcomes during the pandemic in terms of general health, psychological distress and overall QOL, compared with a student reference population measured one year before the pandemic [17]. However, the level of fear of COVID-19 accounted for few of these differences, indicating that other factors such as loneliness and isolation may have reduced nursing students’ QOL [18]. For students involved in clinical practice during the pandemic, concern about infecting others was an additional factor that influenced psychological distress [18].

Consequently, nursing students may be particularly vulnerable during a pandemic. To be able to support nursing students, it is important to get more knowledge about their subjective experiences during the pandemic. Alongside the national survey where we studied fear of COVID-19, general health, psychological distress and overall QOL in nursing students in five universities [18], we also collected qualitative information. In this mixed methods study we included in-depth knowledge combined with the survey. In order to assess psychosocial responses (fear and anxiety) related to COVID-19 we aimed to describe and explore fear of COVID-19, psychological distress, general health and QOL among baccalaureate nursing students at 1 year into the COVID-19 pandemic.

## Methods

### Design and sample

We used a mixed-method design comprising quantitative data from one of the five universities in Southern Norway from the national survey, University of Agder [18], combined with qualitative data from students at the same university. The quantitative and qualitative data were collected sequentially. In the southern part of Norway, the transmission rate was rather low, and the restrictions were not so strict as in other part of Norway. Furthermore, the university could offer the students clinical placement nearly as normal in contrast to other universities where the students experienced disrupted clinical placements or alternative learning arenas. [18].

Between 27 January and 28 February 2021, all baccalaureate nursing students > 18 years of age from the two campuses at University of Agder were invited to take part in the web-based cross-sectional survey, as did students at four other universities in Norway. All baccalaureate nursing students from the same university were invited by the student representatives and the learning portal to participate in focus group interviews on campus[18]. We included students until data saturation was achieved. The focus group interviews took place between 26 April and 6 May 2021. The interviews were arranged in the afternoons and lasted for 50–90 min, and the participants were interviewed face to face. The interviews were moderated by the first author (GR; four groups) or the last author (KH; one group), who are both nurses and professors in health sciences.

### Quantitative data

#### Measures

The survey included questions related to students’ demographics, personal health and study situation during the pandemic; these questions were specifically developed for the national survey by nursing students (including MM), university staff and researchers. Additional measures included four well-validated (nationally and internationally) instruments for assessing fear of COVID-19, psychological distress, general health and overall QOL.

*Characteristics of the respondents* included study site, household status, year of study and age (< 25, 25–29, ≥ 30 years) [18].

*COVID-19 specific questions related to personal health* were developed for the national survey by an expert group consisting of clinicians, nursing students, university staff and researchers [18] and included quarantine history (never, previous, present); feelings of loneliness because of COVID-19 (rated from 1 [strongly disagree] to 5 [strongly agree]); perceived risk for complications of COVID-19 (no, uncertain, yes); and history of suspected, possible or confirmed COVID-19 infection. In the analysis, responses of ‘agree’ and ‘strongly agree’ were pooled under ‘agree.’ To validate the questions, the questions were piloted with nine nursing students (including MM, one of the authors of this study) and the students gave their oral and written feedback. After minor adjustments, a digital pilot study was conducted with 90 physiotherapy students [19]. No adjustments were made after the digital pilot.

*The Fear of COVID-19 Scale (FCV-19 S)* [20] was used to assess fear of coronavirus infection [20, 21]. Seven items (e.g. ‘I am most afraid of the coronavirus’) were rated on a 5-point scale from 1 (strongly disagree) to 5 (strongly agree), with a total score ranging from 7 to 35. On this scale, higher scores indicate a greater fear of COVID-19. In the present study, the average item score was used; it was calculated by dividing the total score by the number of items. The Norwegian version of the instrument has been demonstrated to be reliable and valid in previous studies [20, 21].

*The Hopkins Symptom Checklist (SCL-5)* [22] is available as a Norwegian translation. It includes five items measuring psychological distress (anxiety and depression) that are rated on a 5-point scale from 1 (not at all) to 5 (extremely). We calculated the average item score by dividing the total score by the number of items on the list [23]. Higher scores indicate greater psychological distress. The SCL-5 has been shown reliable and valid [22, 23].

*General health* was assessed using one item from the 36-Item Short-Form Health Survey (SF-36) [24, 25]: ‘In general, would you say your health is: excellent, very good, good, fair or poor?*’* Responses were rated on a 5-point scale ranging from 1 (excellent) to 5 (poor) [26]. Consistent with the SF-36 scoring algorithm, the scale was reverse scored. Thus, higher scores reflect better general health as perceived by respondents. The item was found to be as valid and reliable as multi-item scales also in a Norwegian population [26–28].

*Overall QOL* was rated on an adapted version of the Cantril Ladder on a scale from 0 (not at all satisfied) to 10 (highly satisfied). A score of 6 or more indicates high life satisfaction. The question, ‘All in all, how satisfied are you with your life at this time?’, has been widely used in various populations and in different settings; it is considered a reliable and valid measure of overall QOL [29].

### Qualitative data collection

We used a semi-structured interview guide to ensure inclusion of the issues in focus and to make sure that the moderators covered the same focus. The students were asked questions as the following:

- How has the COVID-19 pandemic influenced your social life?
- How has the COVID-19 pandemic influenced your health?
- How has the COVID-19 pandemic influenced your QOL?

Probing questions were asked to gain deeper insight. To further validate the data collection, the moderators discussed their experiences with the focus group interviews during the interview period. Saturation was reached after five focus group interviews.

## Analysis

The mixed-method analysis strategy represents a parallel mixed data analysis, with separate quantitative and qualitative strands implemented to address related aspects of the research questions regarding the same phenomenon [30]. The findings from both the quantitative and qualitative data collection are integrated in the discussion section.

### Statistical analysis

Descriptive statistics are presented for all students and by year of education. Categorical variables as counts and percentages and continuous variables are described as means (SD). We used the chi-square test for categorical data to reveal associations between categorical variables and analysis of variance (ANOVA) tests for the continuous variables [31].

The analyses were considered exploratory, so no correction for multiple testing was performed. All tests were two-sided, and *p*-values < 0.05 were considered statistically significant. The analyses were performed using IBM SPSS Statistics (version 26) [31].

### Qualitative analyses

We audiotaped the interviews and transcribed them verbatim. The interviews were transferred to NVivo (version 12) software to organise the transcripts into codes and units of meaning. In the analyses, we used a systematic text condensation analysis style [32].

Two researchers (GR and KH) independently read all the material searching for an overall impression and established preliminary sub-themes; the rest of the authors read one interview each and wrote a summary of the overall impression. We then examined the text for units of meaning representing the students’ experiences. In an iterative process, we coded and grouped these units; contrasted and abstracted the content in each group; and finally, discussed and summarised the content of each group into generalised descriptions. All the authors took part in the analysis process and verified the results. To support the analysis, we created mind maps and discussed the analysis at each step to reach an agreement. Quotations were used to illustrate and support the findings [32].

## User involvement

A second-year student (MM) took part in all phases of the quantitative and qualitative study. Furthermore, the student read and contributed to drafts of the paper, and verified the result from a student perspective, which all might be consider increasing the quality of the study. This student is also a co-author of this paper.

## Ethics

The study was conducted according to research ethics guidelines set up in the Helsinki Declaration. The application was approved by the head of department at the university, the Norwegian Center for Research (NSD; Project No 973,745) and the research ethics committee at the faculty of health- and sport sciences (FEK). The students received oral and written information about the study and gave their informed consent to participate.

## Results

### Quantitative data

In total, 396 out of 858 baccalaureate nursing students at University of Agder participated in the survey. Most were living with someone (84%), and 70% were younger than 25 years. Fifty-six per cent reported having felt lonely because of the pandemic. Sixty-two per cent had been engaged in clinical placement during the pandemic, and 83% of the students who had been in clinical practice had cared for patients with confirmed COVID-19 or unclear COVID-19 status. Seventy-two per cent had trust in the way in which their government and 57% in the way University of Agder were handling the COVID-19 situation. Moreover, 70% of the students were concerned about the quality of their education (Table 1).

**Table 1:** Student characteristics of the baccalaureate nursing students at University of Agder for all participants (*N* = 396) and organised by school year

	Total *N* = 396	Year 1 *n* = 142	Year 2 *n* = 127	Year 3 *n* = 127	*p*-values
Years in nursing school					
1	142 (36%)				
2	127 (32%)				
3	127 (32%)				
Age, years					
<25	278 (70%)	112 (79%)	86 (65)	80 (63%)	0.056
25–29	46 (12%)	11 (8%)	26 (20%)	20 (16%)	
≥ 30	72 (18%)	19 (13%)	15 (12%)	27 (21%)	
Living alone					
No	332 (84%)	120 (85%)	108 (85%)	104 (82%)	0.764
Yes	64 (16%)	22 (15%)	19 (15%)	23 (18%)	
Quarantine status related to COVID-19					
Never	236 (60%)	77(54%)	78 (61%)	81 (64%)	0.383
Previous	157 (39%)	63 (44%)	48 (38%)	46 (36%)	
Now	3 (1%)	2 (2%)	1 (1%)	0	
At risk for COVID-19 complications					
No	49 (10%)	16 (11%)	16 (13%)	8 (6%)	0.397
Uncertain	323 (82%)	114 (80%)	103 (81%)	106 (84%)	
Yes	33 (8%)	12 (9%)	8 (6%)	13 (10%)	
Trust in governmental handling of the COVID-19 situation					
Strongly disagree/disagree	36 (9%)	14 (6%)	4 (3%)	18 (14%)	0.016
Neither disagree nor agree	44 (18%)	30 (21%)	29 (23%)	12 (9%)	
Agree	200 (50%)	67 (47%)	68 (53%)	65 (51%)	
Strongly agree	89 (22%)	31 (22%)	26 (20%)	32 (25%)	
Trust in universities’ handling of the COVID-19 situation					
Strongly disagree	18 (4%)	5 (4%)	4 (3%)	9 (7%)	0.080
Disagree	44 (11%)	17 (12%)	17 (13%)	10 (8%)	
Neither disagree nor agree	112 (28%)	37 (26%)	47 (37%)	28 (22%)	
Agree	180 (46%)	67 (47%)	50 (39%)	63 (50%)	
Strongly agree	42 (11%)	16 (11%)	28 (22%)	17 (13%)	
Concern about the quality of education					< 0.001
Strongly disagree	22 (5%)	3 (2%)	6 (5%)	13 (10%)	
Disagree	34 (9%)	6 (4%)	13 (10%)	15 (12%)	
Neither disagree nor agree	64 (16%)	17 (12%)	21 (17%)	26 (21%)	
Agree	142 (36%)	50 (35%)	40 (31%)	52 (41%)	
Strongly agree	134 (34%)	66 (47%)	47 (37%)	21 (16%)	
	2.3 (0.7)	2.5 (0.8)	2.3 (0.7)	2.2 (0.7)	0.026
Feeling lonely due to COVID-19					
Strongly disagree	33 (8%)	8 (6%)	10 (8%)	15 (12%)	0.096
Disagree	61 (15%)	16 (11%)	21 (16%)	24 (19%)	
Neither disagree nor agree	80 (20%)	23 (16%)	28 (22%)	29 (23%)	
Agree	130 (32%)	53 (37%)	40 (32%)	37 (29%)	
Strongly agree	92 (23%)	42 (30%)	28 (22%)	22 (17%)	
Engagement in clinical practice during the pandemic					
Yes	246 (62%)	12 (9%)	117 (92%)	127 (92%)	< 0.001
No	150 (38%)	130 (91%)	10 (8%)	10 (8%)	
Have you been in contact with patients in the following situations during the pandemic?					
Patients with unclear COVID-19 status	148 (61%)	3	64 (55%)	81 (70%)	< 0.001
Patients with confirmed COVID-19 infection	7 (3%)	1	2 (2%)	4 (3%)	
Both (unclear and confirmed)	35 (15%)	0	11 (9%)	24 (21%)	
None	52 (21%)	6	39 (34%)	7 (6%)	

* Categorical data are presented as number (%) and continuous variables as mean (SD). Chi-square tests were used to compare differences in categorical variables and ANOVA tests for continuous data

When comparing students in the three different study years, the most striking differences by February 2021 were that the first-year student had not been in clinical placement, and more students in second year (66%) and third year 3 (94%) had been in contact with patients with confirmed or unclear COVID-19 status. Further, more first-year students (82%) were concerned about the quality of education compared with second-year (70%) and third-year students (62%; Table 1).

## Fear of COVID-19, psychological distress, general health and QOL

For the entire student group, the mean score (SD) for fear of COVID-19 was 2.32 (0.71), that for psychological distress was 1.53 (1.00), that for general health was 3.51 (0.96) and that for QOL was 6.01 (2.06). When comparing first-year students with second- and third-year students, they reported more fear of COVID-19 (2.45 [0.79] vs. 2.28 [0.66] vs. 2.22 [0.70], *p* = 0.026), more psychological distress (1.83 [0.99] vs. 1.43 [0.99] vs. 1.21 [0.66], *p* < 0.001) and lower QOL (5.44 [2.16] vs. 6.19 [2.02] vs. 6.45 [1.86], *p* < 0.001), see Table 2.

**Table 2:** Self-reported fear of COVID-19, psychological distress, general health and overall quality of life among 396 baccalaureate nursing students at the University of Agder

Variables	All (*N* = 396) mean (SD)	1	2	3	P-value^e^‧
Fear of COVID (FCV-19)^a^ (1–5)	2.32 (0.71)	2.45 (0.76)	2.28 (0.66)	2.22 (0.70)	0.026
General health^b^ (1–5)	3.51 (0.96)	3.41 (0.98)	3.59 (1.01)	3.60 (0.96)	0.164
Psychological distress^c^ (SCL-5) (1–5)	1.54 (1.00)	1.83 (0.99)	1.43 (0.99)	1.21 (0.06)	< 0.001
Overall Quality of life^d^ (0–10)	6.01 (2.06)	5.44 (2.16)	6.19 (2.02)	6.45 (1.86)	< 0.001‧

a Higher score of FCV-19 S reflect higher level of fear of COVID-19

b Higher score reflects better perceived general health

c Higher score on Hopkins Symptom Checklist (SCL-5) reflects more psychological distress

d In line with the SF-36 scoring algorithm, the item was reversed. Higher score of overall quality of health reflects better perceived overall quality of life

e ANOVA tests

## Qualitative data

Twenty-three students (7 men, 16 women) took part in the focus group interviews; their age was in the range of 19–32 years, with a mean age of 23 years. The students comprised 10 first-year students, 7 s-year students and 6 third-year students. Most students lived in shared accommodation or with a partner. At the time of the collection of the qualitative data, also the first-year students had been in clinical placement.

**Fig. 1:**
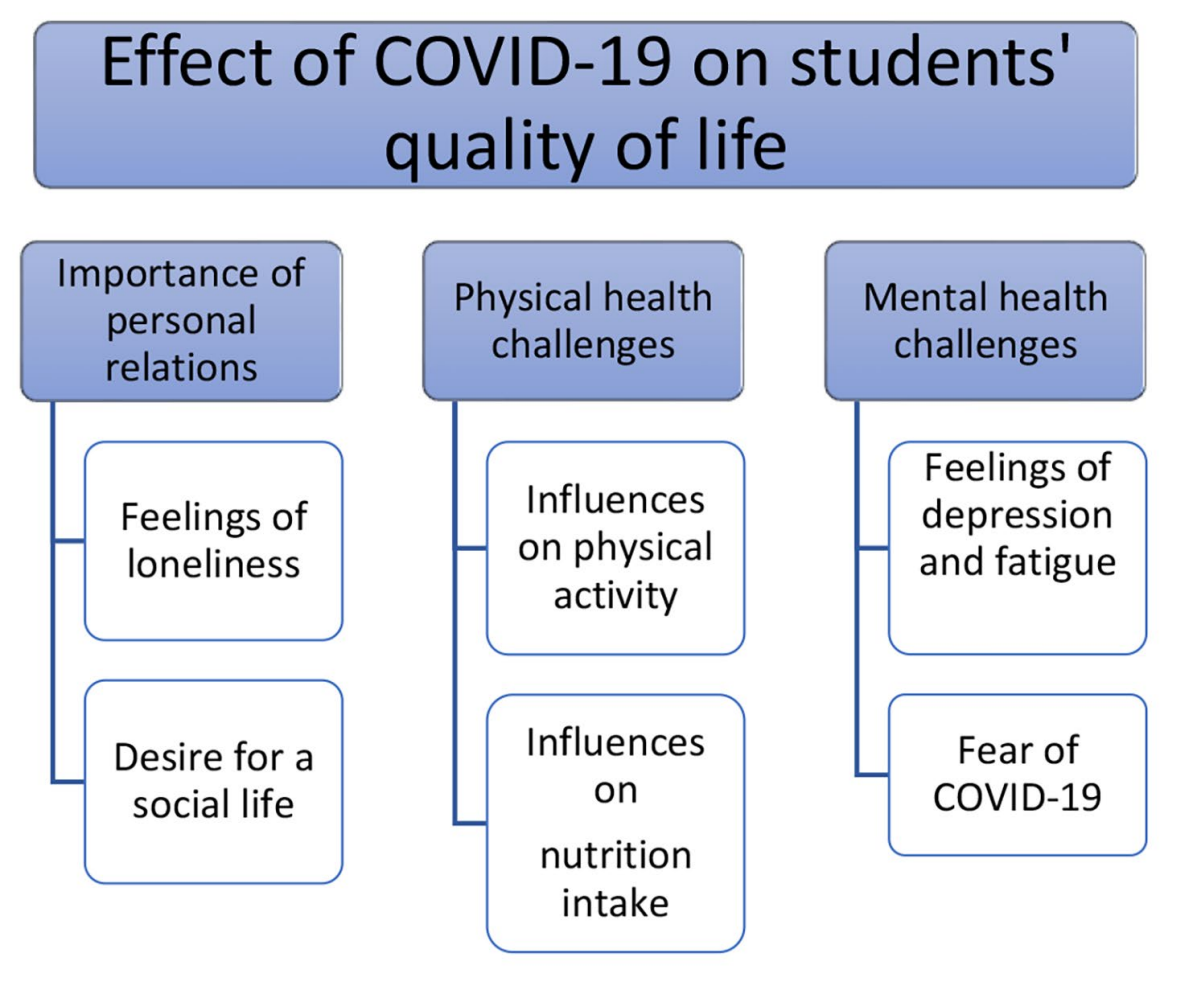
Results from the qualitative analyses with main themes and sub-themes

We identified the overarching theme of *effect of COVID-19 on students’ QOL* and the three following main themes: *importance of personal relations*, *physical health challenges* and *mental health challenges.* Each of the main themes comprised two sub-themes, as listed in Fig. 1.

## Effect of COVID-19 on Students’ QOL

Most of the students felt that the pandemic had influenced their QOL negatively; some suggested that it had reduced their QOL by 20–30%. One of the students commented: *‘I have been unable to do things that bring energy, which is important for my QOL’* (Focus group 3). In contrast, some students experienced high QOL both before and during the pandemic; the difference was the factors important for QOL. In the context of the pandemic, they had learned to focus and appreciate other things and values that they had previously taken for granted. Furthermore, some students organised their everyday lives to improve their QOL; for instance, they would tidy their dwelling or practice given routines. One even commented: *‘I have felt lucky this year. I feel a bit guilty saying I experience a better QOL despite the pandemic’* (Focus group 2).

### Importance of personal relations

comprised the sub-themes of feelings of loneliness and desire for a social life. In general, the participating students described envisioning student life as a time with a socially active lifestyle; however, because of restrictions and lockdowns, this was impossible for them. Several of the students felt lonely; this was especially prominent among the new first-year students because many of them were new in town and had not established contact with other students on campus. It already takes courage, initiative and proactiveness to meet new friends at the university; because of the pandemic restrictions, the arenas for these meetings were different and limited for these students. Some students underlined their responsibility for making friends in due course, while others just continued to feel lonely:

*And now I have to—even if it might feel a bit uncomfortable—randomly ask other students during the programme for semester start if they would like to do something together. I have to dare. I have to work with my mindset, leave my comfort zone, be nice to everyone and make an effort to make friends early.* (Focus group 1)

The students missed meeting one another casually; going to parties; and feeling free, without responsibilities. They missed their extended group of friends. At the same time, a few found it acceptable to live a quieter life without the expectation of being socially active. Pandemic restrictions meant that factors important for QOL were lacking or diminished; for instance, respondents could not meet up with other students or feel free to gather in groups.

In the autumn, when the transmission rate was rather low, the student organisation scheduled an alternative study programme for the beginning of the semester and buddy groups that met the given pandemic restrictions. The buddy groups were crucial to allow new students to become acquainted with others, and some of the new students felt lucky to be with their group or cohort. The importance of the buddy group was also confirmed and emphasised by the more experienced students. One commented, *‘I am a bit embarrassed to say it, but I think I would have felt lonelier if I had been a first-year student’* (Focus group 2). Another stated: *‘I have to say this about the buddy group. We have stuck together. I feel lucky’* (Focus group 1).

Since the transmission rate increased during the winter, some of the students felt they had to select which friends or group they could be with. This was hard, and with changing rules, plans had to be changed or adjusted. Especially during clinical placement, the students felt a responsibility to avoid spreading the COVID-19 virus to patients and other students, which easily could influence their study progression. As a result, they did not see friends, for example, when they went home to visit their parents for weekends or holidays. The students emphasised how they had looked forward to the social part of the student life and recognised that they had lost a lot: *‘I feel like the COVID-19 pandemic has diminished my student life’* (Focus group 2).

Most of the students lived in shared accommodations or with a partner, and they underlined that this was crucial for their social well-being. However, some expressed that it is possible to feel lonely even when living with someone else. This view was especially prominent among those who felt socially isolated due to fear of COVID-19 or limited contacts. They were eager for contact, and some even characterised feelings of desperation in this situation: *‘Most of the time, it is just me and my boyfriend in this apartment 24/7. I feel desperate to see more people’* (Focus group 4).

The restrictions implied that students could only see their families to a limited extent during the semesters. Some students came from a region with a higher transmission rate, and these were especially vulnerable because they were unable to go home. The weeks engaged in clinical placement also meant that students had fewer opportunities to see family and friends. Still, family was important for most of the students’ QOL.

### Physical health challenges

comprised the sub-themes influences on physical activity and influences on nutrition intake. It was found that physical activity was important for the student’s physical health. When the fitness centres closed, students used alternative arenas for exercise and spent a lot of time outdoors, spent time with their pets or went fishing: *‘It is very relaxing just to go fishing’* (Focus group 1). Planning to exercise and stick with a friend helped to fulfil their exercise goals.

Some students felt they had developed a COVID body. They had put on weight, started eating more, developed a habit of eating during the day while attending Zoom tutorials in bed or started eating in a less healthy way. This influenced their body image and their mental health. The students recognised that it was their responsibility to be active and eat properly. In contrast to those who felt less healthy, one student experienced having healthier habits when it was her decision on what and when to eat: *‘I actually eat healthier because I can plan my own meals, deciding what to eat and when’* (Focus group 1).

### Mental health challenges

comprised the sub-themes feelings of depression and fatigue and fear of COVID-19. Several of the students experienced decreased mental health and well-being. Some experienced depressive thoughts, for example, because of catching the virus, worry about spreading the virus or the restrictions the pandemic brought. Others expressed a feeling of fatigue and limited initiative. Students who had experienced mental challenges and depression before the pandemic experienced that the situation influenced their mental health negatively, and some found that it could be hard to get adequate help: *‘It doesn’t help to ask for help when you are unable to receive any’* (Focus group 3).

The students found it exhausting to adapt and follow the changing restrictions and rules. As nursing students, they were more aware of the mental health in general and the mental health of their fellow students. One student reported that she had been more attentive towards friends, sending extra text messages or calling them. Some students expressed that the pandemic period was one of the worst times in their lives, and they hoped that the future would be better.

When students engaged in regular physical activity, it positively influenced their mental health. The students spent time with their cohort, and when they were not allowed to meet indoors, they went outdoors and enjoyed nature together: *‘If you are active and use your body, it will positively influence your mental health’* (Focus group 5).

Several of the students said they had worked with their mindset and mental approach to life during the pandemic. The pandemic had taught them to take care of their health, although it required discipline to manage their study and everyday activities.

## Discussion

When we explored fear of COVID-19, general health, psychological distress and quality of life among baccalaureate nursing students at 1 year into the COVID-19 pandemic, we found that the pandemic influenced the students’ QOL. The pandemic reduced the students’ opportunities to live an active (student) life; many felt lonely and missed having social relations. The students experienced that this negatively influenced their QOL, physical health, and especially, their mental health. The challenges were most prominent among first-year students. However, the students also developed strategies for maintaining a social life to increase their physical and mental health and QOL under the given COVID-19 restrictions. Inferences of the quantitative and qualitative results are integrated into meta-inferences in the discussion section.

The quantitative data showed that the students reported general health and psychological distress at a level that was comparable with the pre-pandemic level, whereas their level of QOL differed during the pandemic [18]. Most students experienced their QOL was influenced negatively, mainly because their previously known factors for QOL were lacking or had changed [33]. Previous studies have identified increased stress, anxiety [8–10], loneliness and mental health problems among nursing students because of the COVID-19 pandemic [10–13]. However, some of our students also revealed resilience as the pandemic went by, for example, by focusing on other issues that brought better QOL. Resilience during the pandemic has been seen in longitudinal studies of the general population; it is characterised as a surprising ability to adapt [6]. In the region of the present university, the students have access to woodlands and outdoor spaces, and there are arenas available for all people (including students); this made it easier for them to choose alternative activities. Despite signs of resilience and adaptation among most students, as well as that QOL among the students at the studied university was higher than levels reported in other universities taking part in the national survey [18] (see also supplementary Table 2), most of the students experienced reduced QOL because of the pandemic.

About 50% of the students in the present study felt lonely because of the pandemic. They missed personal relations because they were unable to meet with other students, their friends, or their families. A previous study identified a correlation between feelings of social isolation and mental health among nursing students [34]. It has also been found that students struggle to implement knowledge when they feel lonely [35]. Our first-year students reported being lonelier and more vulnerable thanthesecond-and third-year students did, probably because of their limited opportunities to establish an educational and social network. The yearly programme for starting the autumn semester at the university is an event most students look forward to. At this time, the students connect with one another, and the university arranges buddy groups focused on helping new students to meet other students. However, this annual event had to be arranged with restrictions and adaptations because of the pandemic, and some of the initial intentions could not be realized.

The students conveyed that the feeling of missing personal relations with other students became a stressor for them. Social and academic isolation can have major consequences when acquiring professional knowledge [10, 35, 36]. Such findings were also reported in another study based on a national sample where the nursing students showed strong feelings of social and academic isolation during the pandemic [37].

In our study, some of the students seemed to develop coping strategies to feel less lonely in finding smaller groups or cohorts to support them. Furthermore, clinical placements were described as an important place to connect to other students during the pandemic, as well as helping them to establish structure in their everyday lives, which was good for implementing predictability. That the present university could provide clinical placement nearly as normal was beneficial for the students. Previous studies showed that students experienced uncertain and stressful environments and struggled with their learning outcomes during COVID-19 [35]. To support students during a crisis like the pandemic, it is important for the university to create options to maintain distance counselling services for all students. Moreover, universities should develop strategies for identifying students who need psychosocial support and facilitating contact with supervisors and space to unwind [35].

The students in our study reported more fear of COVID-19 than the general population did [21], and the qualitative data revealed worsened physical and mental health among some of the students[4], including depressive thoughts, fatigue and lack of energy. These findings were especially prominent among first-year students. Nursing students are most likely aware of general advice for better mental and physical health [38]. Given the COVID-19 restrictions, the students had a greater extent to self be responsible and chose activities that bring better health. The free time that came about as a result of the restrictions may have given the students the opportunity (or forced them) to make more conscious choices and perhaps explore former underused or new ways to increase their physical and mental health [39].

The students’ health seems to have been influenced by the COVID-19 restrictions, and this may have caused more stress and fear. Such findings have also been seen in other studies among nursing students, who report increased levels of stress, anxiety, health problems, sleeplessness, and fear about their study progression [5, 8–11]. The results from a systematic review suggest that providing regular mental health assessments or online mental health services to students may increase their well-being in situations like a pandemic [40]. Ultimately, empowering students to cope with challenging emotions and thoughts might contribute to the development of both individual students and the profession as a whole [41].

## Strengths and limitations

The strengths of the current study are the inclusion of both qualitative and quantitative methods. It is a strength that we used well-validated instruments to measure psychological distress, physical and mental health and QOL. The qualitative analysis was conducted by authors who are experienced nurse educators, which may ensure knowledge of the context; however, it may also represent a bias in the data analysis. To minimize the bias, we tried to put our preconception aside. Furthermore, we used probing questions to invite the students to elaborate their experiences. All authors took part in the analysis process, which strengthened the credibility of the study. Thematic synthesis allowed different perspectives and contexts to be explored. To ensure transparency, the different steps of the analysis were specified and followed [32]. The discussion and reflection between the researchers about the analysis and findings of both data sources strengthened the trustworthiness of the insights gained. Further, user involvement and collaboration with students is a strength in this study. Students were involved in several stages of the process and validated the themes and findings.

One limitation was the cross-sectional nature of the survey, which allowed it to reveal only statistically significant associations between the variables and did not allow conclusions to be drawn about causality. Furthermore, we have no information about the students who did not take part in the survey.

## Conclusion and implications

The COVID-19 pandemic negatively influenced nursing students’ QOL and physical and mental health, and it increased their feelings of loneliness. However, most students who participated in this study also adapted strategies and resilience factors to cope with the situation. The students may have learned additional skills and mental mindsets that can be useful in their future professional lives. In future pandemics, it might be considered important for universities to take on responsibilities for supporting students’ mental health, physical health and QOL.

## Electronic supplementary material

Below is the link to the electronic supplementary material.

Supplementary Material 1

## Data Availability

The datasets used and/or analyzed during the current study are not publicly available due to General Data Protection Regulation laws but are available from the corresponding author on reasonable request and with permission from the Norwegian Centre for Research Data.
