# Supplementary material for: Being a nursing student during the coronavirus pandemic: a mixed methods study
Source: BMC Nurs. 2023 Mar 3;22:60. doi: 10.1186/s12912-023-01218-8 (PMC9982793; doi:10.1186/s12912-023-01218-8)
Supplement: Supplementary file 1 — Supplementary Material 1 [file 12912_2023_1218_MOESM1_ESM.docx]

Supplementary table 1. Comparing students from University of Agder with the four other universities in the national survey (19)

|  | University of Agder  N=396 | Other universities  N=2310 | p-value |
| --- | --- | --- | --- |
| Years in nursing school    1    2    3 | 142 (36%)  127 (32%)  127 (32%) | 933 (42%)  674 (31%)  603 (27%) | 0.043 |
| Age, years    <25    25-29    ≥ 30 | 278 (70%)  46 (12%)  72 (18%) | 1567 (71%)  551 (15%)  310 (14%) | 0.037 |
| Living alone    No    Yes | 332 (84%)  64 (16%) | 1797 (82%)  403 (18%) | 0.179 |
| At risk for COVID-19 complications    No    Uncertain    Yes | 40 (10%)  323 (82%)  33 (8%) | 287 (13%)  1766 (80%)  156 (8%) | 0.213 |
| Trust in governmental handling of the COVID-19 situation    Strongly disagree/disagree    Neither disagree nor agree    Agree    Strongly agree | 36 (9%)  71 (18%)  200 (51%)  89 (22%) | 195 (9%)  491 (22%)  1144 (52%)  380 (17%) | 0.080 |
| Trust in universities’ handling of the COVID-19 situation    Strongly disagree    Disagree    Neither disagree nor agree    Agree    Strongly agree | 18 (4%)  44 (11%)  112 (28%)  180 (56%)  42 (11%) | 163 (7%)  403 (18%)  671 (30%)  802 (36%)  171 (8%) | <0.001 |
| Concern about the quality of education  Strongly disagree    Disagree    Neither disagree nor agree    Agree    Strongly agree | 22 (5%)  34 (9%)  64 (16%)  142 (36%)  134 (34%) | 72 (3%)  (126 (6%)  223 (10%)  727 (33%)  1062 (48%) | <0.001 |
| Feeling lonely due to COVID-19    Strongly disagree    Disagree    Neither disagree nor agree    Agree    Strongly agree | 33 (8%)  61 (16%)  80 (20%)  130 (33%)  92 (23%) | 131 (6%)  319 (14%)  364 (16%)  769 (35%)  626 (28%) | 0.048 |
| Engagement in clinical practice during the pandemic    Yes    No | 246 (62%)  150 (38%) | 1345 (61%)  864 (39%) | 0.343 |
| Have you during the pandemic been in contact with patients with the following situation?  Patients with unclear COVID-19 status  Patients with confirmed COVID-19 infection  Both (unclear and confirmed)  None of them | 148 (61%)  7 (3%)  35 (15%)  52 (21%) | 664 (51%)  44 (4%)  203 (15%)  389 (30%) | 0.017 |

The number of students will vary due to some students lack clinical practice during the pandemic period

Supplementary table 2. Self-reported fear of COVID-19, general health, psychological distress and overall quality of life among 396 baccalaureate nursing at University of Agder compared to the four other universities in the national survey

| Variables | University of Agder  N=396  mean (SD) | Other universities  N=2310 | P-value*‧ |
| --- | --- | --- | --- |
| FCV-19^a^ (1-5) | 2.32 (0.71) | 2.47 (0.81) | <0.001 |
| General health^b^  (1-5) | 3.51 (0.96) | 3.50 (0.93) | 0.547 |
| Psychological distress^c^  (SCL-5) (1-4) | 1.54 (1.00) | 1.71 (1.00) | 0.003 |
| Overall Quality of life^d^  (0-10) | 6.01 (2.06) | 5.41 (2.16) | <0.001 |

a Higher score of FCV-19S reflect higher level of fear of COVID-1

b Higher score reflects better perceived general health.

c Higher score on Hopkins Symptom Checklist (SCL-5) reflect more psychological distress

d In line with the SF-36 scoring algorithm, the item was reversed. Higher score of overall quality of health reflects better perceived overall quality of life.
